# Age- and sex-related changes in vertebral trabecular bone architecture in Neolithic and Mediaeval populations from Poland

**DOI:** 10.1038/s41598-024-59946-z

**Published:** 2024-05-01

**Authors:** Francesco Maria Galassi, Wiesław Lorkiewicz, Jarosław Filipiak, Anna Nikodem, Elżbieta Żądzińska

**Affiliations:** 1 Department of Anthropology, Faculty of Biology and Environmental Protection, University of Lodz, Lodz, Poland; 2 Department of Mechanics, Materials and Biomedical Engineering, Faculty of Mechanical Engineering, Wroclaw University of Science and Technology, Wrocław, Poland; 3 Biological Anthropology and Comparative Anatomy Research Unit, School of Medicine, University of Adelaide, Adelaide, SA 5005 Australia

**Keywords:** Anthropology, Bone, Osteoporosis, Prehistoric, Trabeculae, Trabecular architecture, Anthropology, Archaeology, Anatomy, Medical research

## Abstract

This paper investigates trabecular bone ontogenetic changes in two different Polish populations, one prehistoric and the other historical. The studied populations are from the Brześć Kujawski region in Kujawy (north-central Poland), one from the Neolithic Period (4500–4000 BC) and one from the Middle Ages (twelfth-sixteenth centuries AD), in total 62 vertebral specimens (32 males, 30 females). Eight morphometric parameters acquired from microCT scan images were analysed. Two-way ANOVA after Box-Cox transformation and multifactorial regression model were calculated. A significant decrease in percentage bone volume fraction (BV/TV; [%]) with age at death was observed in the studied sample; Tb.N (trabecular number) was also significantly decreased with age; trabecular separation (Tb.Sp) increased with advancing age; connectivity density (Conn.D) was negatively correlated with biological age and higher in the Neolithic population. These data are found to be compatible with data from the current biomedical literature, while no loss of horizontal trabeculae was recorded as would be expected based on modern osteoporosis.

## Introduction

Bone loss with increasing age in adulthood has been shown to be a universal phenomenon in past human populations, as confirmed by the assessment of the osteoarchaeological record^1,2^, hence offering a valuable comparative model for the current epidemiology and morphology of osteoporosis worldwide.

However, while the antiquity of osteoporosis is not in question, some striking differences have so far been noted between investigated ancient and contemporary populations, most notably a lesser degree of postmenopausal bone loss in females and a higher risk in younger individuals for both sexes^3,4^. Such discrepancies have been considered both in light of the investigated populations’ historical record and on the basis of diverging research protocols and preservation conditions (also assessing diagenetic influence)^2^. Indeed, dual-energy X-ray absorptiometry has been widely used in biomedical studies on contemporary populations and has so far also been applied to ancient osteological remains on account of its being considered a surrogate marker of bone strength^5^.

A previous study by our group^6,7^—considering the predicted increase in incidence of osteoporosis and fragility-related fractures in contemporary male individuals^8^—applied this methodology to focus on the evaluation of patterns of age-related bone loss (based on bone mineral density—BMD) and osteoporosis-related fractures in males from the Kujawy region in north-central Poland, spanning a period of 6000 years from the Middle Neolithic to the Early Modern Age^6^. The analysis showed that age-related bone loss in both sexes is substantially rather similar to the patterns observed in modern-day Western populations but differed in that the studied ancient male populations appeared to be at a higher risk of osteoporotic fractures occurring earlier and more frequently than in females from the same population^6^. Remarkable sex difference in BMD were also a very notable result: in both periods (Neolithic and historic times) males showed higher BMD values than females, especially in the younger age classes. Even more pronounced differences occurred between the analysed periods: both males and females had much higher BMD values in the Neolithic compared to the historic times. We considered that one of the reasons for these differences was the much higher workload of the Neolithic people compared to historical populations. However, a more complete interpretation of the specificity of bone maintenance in these populations requires going beyond a quantitative parameter such as bone mineral density.

Another analysis by Lorkiewicz et al.^9^ indicates that the females representing populations from Poland dated from 4500 BCE to the early nineteenth century CE (i.e. skeletal series that are part of the osteological collections of the Department of Anthropology of the University of Lodz and the Lodz Museum of Archaeology and Ethnography) with a high degree of parturition pits (related to obstetrical events), regardless of age at death, were characterised by higher bone mineral density than the females without such pits.

Nonetheless, as a general note, it should be underlined that, while it is still considered to be the diagnostic gold standard, dual-energy X-ray absorptiometry assessing BMD only accounts for 60% of bony fragility variation^5^. Indeed, BMD does not fully explain ontogenetic changes in trabecular architecture, tissue properties and accumulation of microdamage, as it has been shown that a patient’s fracture risk is also—and paramountly—influenced by bone quality^10^. This is especially evident when one considers that a parameter like loss of strength is more affected by perforation of the trabeculae than by their general thinning^5^.

These observations, both methodological and interpretative, are in line with the original observation by Albright and colleagues, part of a study focused on the links between menopause and osteoporosis and their clinical interplay, that osteoporosis is caused by deficient osteoblastic activity, hence it can be defined a deficit of formation rather than mineralisation^11,12^. Most subsequent research on osteoporosis was modelled on this realisation, especially thanks to the introduction of CT scan and microCT analysis, the latter being nonetheless not allowed on living patients due to high radiation exposure, yet excellent for in vitro or cadaver studies^5^.

Trabecular bone architecture (and its changes with age) is most often studied as one of the main features which determine bone’s ability to resist fracture, i.e. bone quality^1,13^. Ex-vivo cadaveric studies of the trabecular architecture focus mainly on areas of the skeleton which are important for the occurrence of osteoporotic fractures: the proximal femur, distal radius and vertebral bodies^14^. Since spinal compression fractures are the most common osteoporotic fractures^15^, a particularly vast number of published studies concern the trabecular architecture of the vertebrae, as well as the changes it undergoes with the normal aging process and in the context of osteoporosis—which can be considered as ultimate manifestation of this process^16^. These changes include a decrease in relative bone volume and trabeculae number, loss of trabeculae connectivity, an increase in trabecular separation and structural anisotropy (as the consequence of preferential loss of horizontal trabeculae), and a shift from plate-like trabeculae to more rod-like structures^14,17–19^. Contradictory results are presented in literature in the case of age-related changes in trabecular thickness (reviewed in Thomsen et al., Hulme et al. and Chen et al.^17,19,20^, amongst others). It appears that the best explanation for this phenomenon was provided in a detailed 3D histomorphometric analysis by Thomsen and co-authors^21^, who indeed demonstrated a significant decrease in the horizontal to vertical trabecular thickness ratio due to relatively more pronounced thinning of horizontal than the vertical trabeculae with age (with no age-dependent changes in thickness of vertical and horizontal trabeculae analysed separately).

Different results were also obtained with regard to sex differences in trabecular bone architecture and its changes with age. Many studies indicate “better” (in terms of bone resistance to fracture) characteristics of trabecular bone quality in men compared to women of the same age, as well as sex-dependent rate of deterioration of trabecular bone with aging, which occurs faster in women than men, especially at and after the menopause^14,19,22^. Other authors found that there is little or no effect of sex on vertebral trabecular bone architecture parameters and their changes with age, even when at the same time such differences occurred in other sites of the skeleton examined^16,23,24^.

Vertebral trabecular bone architecture has also been studied in a number of prehistoric and historical populations, e.g. Early Bronze Age population from Austria^25^, Mediaeval Nubian population^26^, Imperial Roman population from Italy^2^, Mediaeval rural (Wharram Percy) and urban (London) populations from England^4,27^, and Early Modern population from 18 to 19th London^28^. The authors of these studies found a generally similar direction of changes with age in the quantity and quality of cancellous bone as in modern populations. However, there were also some significant differences. The most important one concerned the age at which increased bone loss was observed in women. In some populations, especially in the Wharram Percy one, females experienced significant trabecular bone loss earlier than today, with no major changes later in life, in the postmenopausal period. This pattern of trabecular architecture changes with age was explained by reproductive behaviour specific for Mediaeval rural population, i.e. high parity and extended periods of lactation^27^. On the other hand, greater loss in bone structure in young females compared to later age groups was also found in an urban population living in early modern London. In other samples, such as the Bronze Age population from Austria and Mediaeval population from London, women had a pattern of bone loss similar to that seen in modern populations^4,28^. Sex differences in trabecular bone volume and structure and their age-related changes were also different in the studied populations. Most of them showed the pattern considered typical of modern populations, with greater postmenopausal and age-related trabecular bone loss in women than in men. However, in the Wharram Percy population there were no statistically significant sex differences in any of the parameters examined. The authors explained this by the similar involvement of both males and females in hard labour activities related to the farming lifestyle^27^.

Bioarchaeological studies often demonstrate generally better bone quantity and quality in the past compared to modern populations, despite numerous reports of the relatively poor health status of past populations, resulting, for example, from insufficient nutrition. It is argued that this is the result of two factors: high parity and physical activity. Many clinical studies show that pregnancy and lactation lower BMD due to increased skeletal resorption of calcium resulting from substantial transfer of this mineral from mother to foetus and infant^29–35^, but that decrease is only temporary^36–40^, or even contributes to higher BMD values throughout the lives of women who had children, with the positive effects increasing with the parity status^41–43^. Such results were referred to when considering high parity in the past populations as a possible cause of the observed tendency to early bone loss in young women with generally better bone maintenance in this sex in archaeological populations compared to modern ones^4,9,44^. Greater physical activity is often cited as another important factor causing higher BMD and better trabecular bone properties as well as less severe bone loss with aging in prehistoric and historical populations^6,27,45,46^. One may ask whether such diachronic differences in trabecular bone architecture as between archaeological and modern populations also occurred in the past. Populations that are temporally diverse but come from the same geographic area are particularly useful to answer such a question. In the present paper we analyse microCT scan data from vertebral samples to assess the presentation and degree of age- and sex-related changes in trabecular bone architecture in Neolithic and Mediaeval populations from Kujawy, north-central Poland. Based on the above literature review and the results of our previous studies on BMD in these populations, we formulated the following hypotheses:

1. The vertebral trabecular bone architecture in both populations generally underwent the same changes with age as in modern epidemiological studies and in archaeological populations described so far.
2. The Neolithic population was characterised by better (in terms defined above) vertebral trabecular bone architecture than the Mediaeval population.
3. In both analysed populations, trabecular bone architecture was influenced by sex. We expect relatively better trabecular bone quality in men than in women, as was the case in our previous BMD studies (a denser bone microarchitecture should be reflected in a higher BMD values)^47^. However, the opposite direction of sex differences in trabecular bone architecture may also be expected considering the above-mentioned higher frequency of osteoporotic fractures in men in these populations. Moreover, some studies show no significant relationship between BMD and microstructural parameters of the trabecular bone of the lumbar spine after adjustment for age and body mass^48^.

## Materials and methods

### Bioarchaeological sample

The research included the fourth lumbar vertebrae of 62 adult specimens, 32 males and 30 females, representing the populations from the Brześć Kujawski region in Kujawy (north-central Poland) from the Neolithic (4500–4000 BC) and Mediaeval (twelfth–sixteenth centuries AD) periods. All skeletons from which the vertebrae were sampled were macroscopically examined for pathological changes. Only vertebrae without advanced external lesions that could affect the analysed trabecular bone parameters were selected for microCT examination. Despite the large skeletal series examined it was not possible to completely exclude pathological specimens from the analysis. This concerned mainly older individuals, but in their case changes of this type (still of a moderate degree) can be considered a normal part of the aging process.

The sex of the individuals was determined primarily using pelvic morphology, including the features of the pubic region^49^, subpubic arch angle^50^, pubic length/ischium length ratio^51^, the width and shape of the sciatic notch^16,51,52^, and the presence/absence and the form of the preauricular sulcus of the ilium^53–55^. In case of poor preservation of pelvic bones, the cranial traits were also analysed as described by Buikstra and Ubelaker^53^. Age at death of the individuals was estimated based on the evaluation of changes in pubic symphysis and in the sternal ends of the ribs, ectocranial vault suture obliteration, and tooth wear^56–60^. Moreover, the final stages of skeletal ossification (secondary centres in the sternal ends of the clavicles, lower vertebrae, and ilia) were analysed in the case of the youngest age group. The age of each individual was determined based on all criteria available in the given case (this comprehensive approach was intended to avoid comparing individuals whose biological age was determined based on single criteria that could produce divergent results). In the case of tooth wear, a different approach was used for the Neolithic and Mediaeval skeletons due to higher rate of tooth wear observed in individuals from the Neolithic population. For Neolithic individuals, charts published by Brothwell were used^61^. For Mediaeval skeletons, we used the methods proposed by Mays and co-workers^60^. The age of the specimens was assigned mostly in 10-year age classes, but also 15-year or even 20-year classes were used for some older individuals. The age of the oldest individuals was only defined as 50 + or 60 + years due to the limitations of the skeletal methods of age-at-death estimation used in bioarchaeology^62^. In these cases, 80 years, i.e. longevity in contemporary Poland (link: https://stat.gov.pl), were adopted as the upper limit of the age class, assuming that this trait is relatively invariant in human populations, including past ones^63,64^. In multiple regression analysis, the middle values of these age classes were used as proxies for the actual age of the individuals. In other analyses (descriptive statistics, two-way ANOVA), individuals were classified as young adults (18–29 years), middle adults (30–49 years), and older adults (50 + years). Such classes were widely used by other authors in papers on osteoporosis in archaeological populations^3,65^.

There are many studies that indicate that body size and mass can affect trabecular bone structure. For this reason, we included stature (which is the most accurate component of body size to reconstruct in archaeological populations) as one of the independent variables explaining the variability of trabecular bone parameters in the multiple regression analysis^66–69^. The stature was calculated from the femora using Pearson’s formulas^70^.

**Figure 1:**
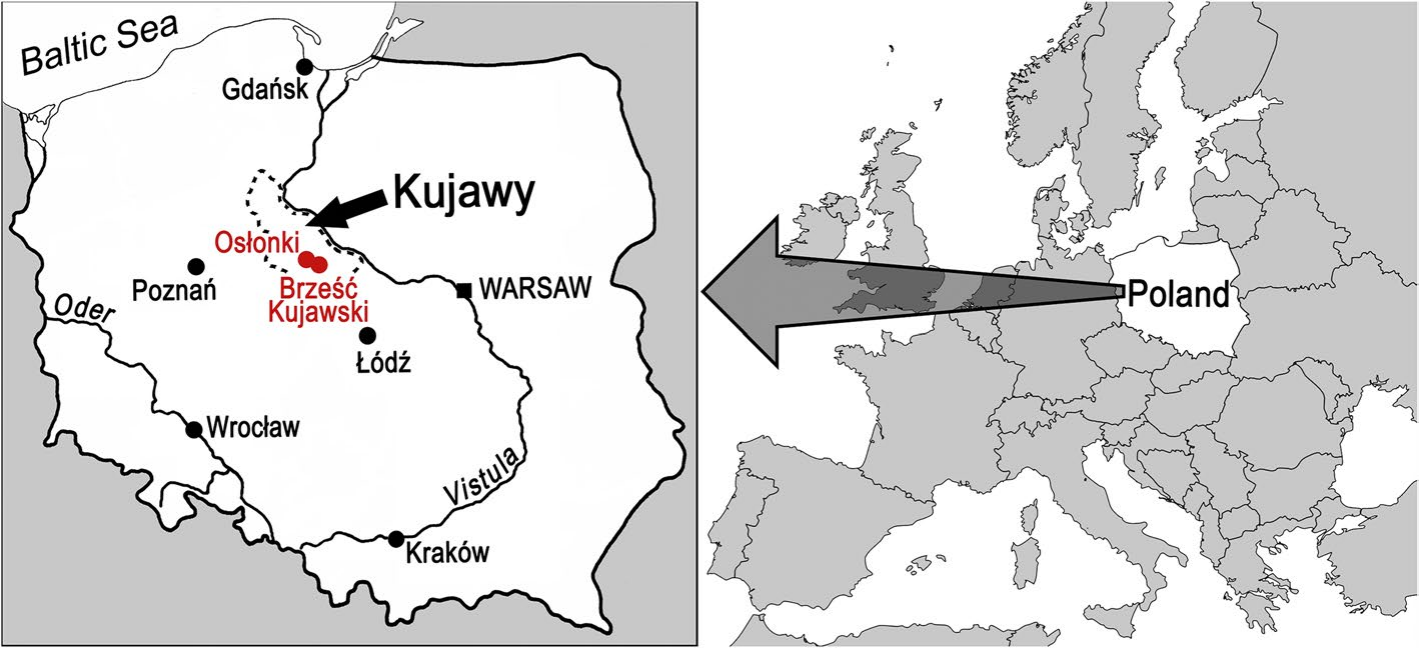
Position of Brześć Kujawski (Latitude: 52.60628, Longitude: 18.90129) and Osłonki (Latitude: 52.61622, Longitude: 18.78584), from where the sample originated, on the geographical map of Europe. The sites are located in the Kujawy (Eng. Kuyavia) region in north-central Poland.

The distribution of the examined individuals by biological sex and age is presented in Table 1. The Neolithic skeletons come from cemeteries located within two large settlements: Osłonki (Osłonki, site 1) and Brześć Kujawski (BK4 and BK5 sites), which existed at the same time, about 10 km far from each other (Fig. 1). The Neolithic samples represent the Brześć Kujawski Group of the Lengyel Culture (BKG), which was, in this part of Europe, the final stage of development of a cultural tradition initiated by the Linear Pottery Culture in the middle of the 6th millennium BCE^71,72^. BKG communities consisted of sedentary agropastoral farmers, genetically being an amalgamation of allochthonous farmers of Near Eastern origin and indigenous hunter-gatherers^73–75^. The Mediaeval sample represents a typical indigenous population from central Poland from that period, inhabiting a large settlement, probably of a partially rural nature^76^. The skeletal series are part of the osteological collections of the Department of Anthropology, University of Lodz, and of the Museum of Archaeology and Ethnography in Lodz. They were stored and investigated in accordance with the Polish regulations on archaeological human remains^77^.

**Table 1:** Distribution of studied specimens according to sex and age.

Period/Series	Sex	Age group (in yrs)			Total
		18–29	30–49	50 +	
Neolithic: Osłonki, BK4 and BK5	Male	4	5	3	12
sites (4500–4000 BC)	Female	3	2	2	7
Mediaeval: SBK4 site (12–16th cent. AD)	Male	8	5	7	20
	Female	7	10	6	23
Total		22	22	18	62

### Trabecular architecture analysis

All micro-CT images were acquired by 1172 SkyScan, Bruker (Kontich, Belgium) with conical beam, operated with 0.1 mm mm Cu filter at 100 kV and 100 μA, respectively, and a 1000 × 524 pixels CCD camera. The sample was rotated from 0° to 180°, in steps of 0.35°.

**Figure 2:**
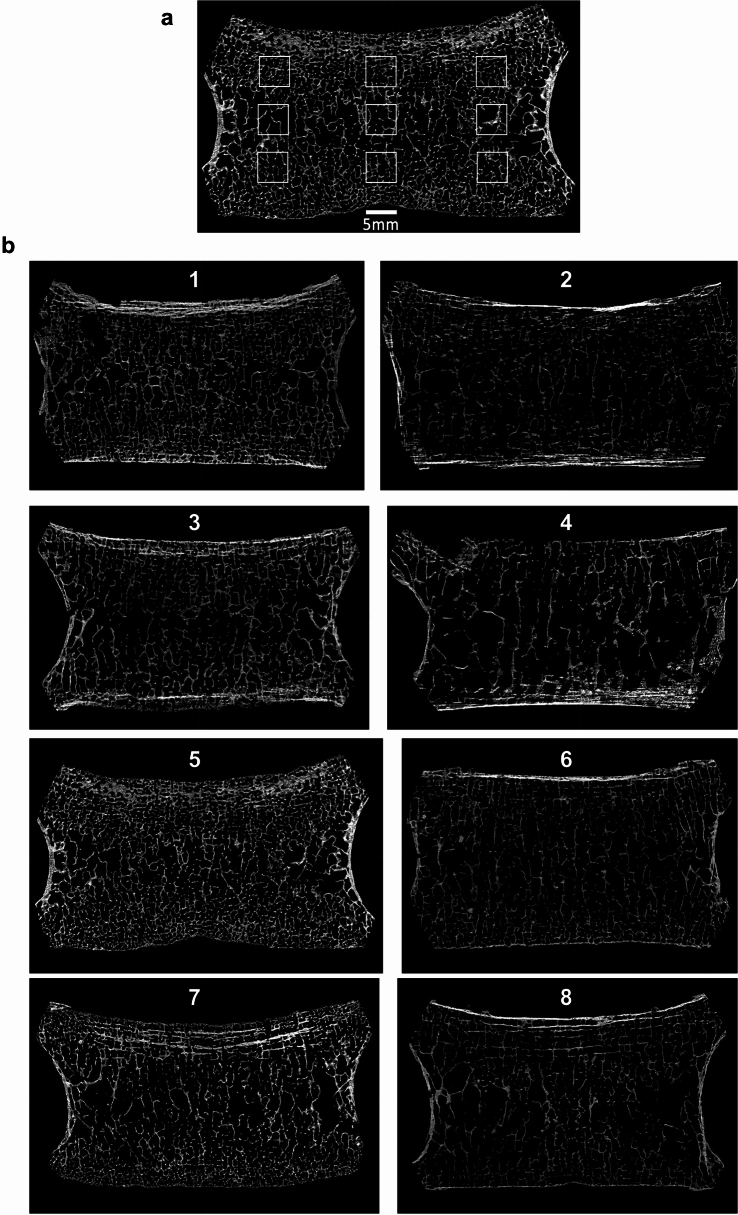
(**a**) Frontal section of the vertebral body with the location of the 5 × 5 × 5 mm cubic volumes of interest (VOI—not to scale), for which the structural parameters analysed in the study were calculated (2D image). (**b**) Coronal cross-sections of L4 vertebral bodies from one younger and one older male and female from both populations analysed (with given values of bone architecture parameters, which showed a significant relationship with age). (b1) Neolithic, male, age class 18–29 (BV/TV 19.883; Tb.Sp 0.679; Tb.N 1.126; Conn.D 9.119). (b2) Neolithic, male, age class 50 + (BV/TV 11.312; Tb.Sp 0.816; Tb.N 0.780; Conn.D 7.103). (b3) Neolithic, female, age class 18–29 (BV/TV 19.447; Tb.Sp 0.842; Tb.N 0.970; Conn.D 5.979). (b4) Neolithic, female, age class 50 + (BV/TV 11.459; Tb.Sp 1.123; Tb.N 0.650; Conn.D 5.883). (b5) Mediaeval, male, age class 18–29 (BV/TV 18.868; Tb.Sp 0.587; Tb.N 1.281; Conn.D 12.278). (b6) Mediaeval, male, age class 50 + (BV/TV 11.851; Tb.Sp 0.795; Tb.N 0.782; Conn.D 5.441). (b7) Mediaeval, female, age class 18–29 (BV/TV 14.230; Tb.Sp 0.763; Tb.N 0.941; Conn.D 6.339). (b8) Mediaeval, female, age class 50 + (BV/TV 9.681; Tb.Sp 0.956; Tb.N 0.651; Conn.D 3.651).

As some vertebrae showed local postmortem damage to the trabecular structure or the presence of mineral sediment inside the vertebral body that made it impossible to examine the entire cross-section, the analysis was based on nine volumes of interest (VOIs). For this purpose, a virtual 5 mm thick coronal slice was selected in the midbody of the vertebrae (calculated in the middle of the vertebral body between its periosteal contour along the sagittal axis). From this slice, a central region was selected that covered 75% of the height and 75% of the width of the vertebral body. Since some of these regions included damaged trabecular bone, nine smaller 5 × 5 × 5 mm sections (VOI’s) were selected from them according to the scheme: one in the middle and other eight at the extremes (and the “extremes” were the edges of the previously selected “75%” region) (Fig. 2a–b**;** VOIs Ia-IIIc). The positions of individual VOI’s were thus scaled to the size of a given vertebral body, which is important due to the visible (and well documented in the literature^5,17,78^) differences in the structure of trabecular bone in the entire cross-sections. This allowed us to avoid concentration of VOI’s in the center of the cross-section in the case of larger vertebrae (where the trabeculae were thicker and sparser) and their shifting to the more outer areas of the cross-section in the case of smaller vertebrae (where the trabeculae were thinner and denser). A section thickness of 5 mm was chosen following the work of other authors^3,4,27^ on trabecular bone architecture in archaeological samples, and the 5 × 5 × 5 mm VOI’s as a compromise between avoiding diagenetically altered areas and choosing the largest possible undamaged sections. The values of the analysed parameters for a given individual were calculated as the arithmetic mean of the values obtained separately for each of the nine VOIs. For eight individuals one VOI in the left or right columns could not be obtained due to postmortem damage to this part of the vertebra (two lacked VOI Ib, four VOI IIIb, and two VOI IIIc). Because there were no statistically significant differences in the analysed parameters between the VOIs in the same rows from columns I and III, the missing sample was replaced with data for VOI from the opposite side (i.e. Ib was replaced by IIIb, IIIb by Ib, and IIIc by Ic).

This approach made it possible to increase the number of vertebrae included in the analysis compared to the one using the entire vertebral body cross section. Eight morphometric parameters were measured using the SkyscanTM CT-analyser software in the selected sections (the unit of measurement is given in square brackets; the lack of this information indicates the unitless values of the parameter): (1) Bone volume fraction (BV/TV; [%]) indicates the ratio of the trabecular bone volume to the total sample volume. (2) Trabecular thickness (Tb.Th; [mm]) indicates the average thickness of the trabeculae. Since some data from literature indicate that mean trabecular thickness decreases with age, the expected result is a decrease in value of this parameter between younger and older age groups^16,20,79^. (3) Trabecular separation (Tb.Sp; [mm]) indicates the mean distance between trabeculae. (4) Trabecular number (Tb.N; [mm-1]) indicates the average number of trabeculae in a sample volume. This parameter is directly related to the two previous ones and is computed by microtomography software as inverse of sum Tb.Th and Tb.Sp. (5) Trabecular pattern factor (Tb.Pf; [mm-1]) indicates relative convexity or concavity of the total trabecular bone surface in the sample, where concavity indicates the presence of nodes (connectivity of trabeculae), and convexity indicates disconnected struts. Lower values of this parameter are interpreted as indicating better connected trabecular structure while higher ones as a more disconnected trabecular bone. Although this interpretation of Tb.Pf has been questioned, the parameter is still often used to assess the quality of trabecular bone^80^. (6) Structure model index (SMI) indicates the relative prevalence of rod-like (higher index values) or plate-like (lower index values) trabeculae in the sample, whose interpretation was followed in this study as indicated in several publications, yet there exists criticism of this index and the proposal to use instead the mean ellipsoid factor (EF) to measure rods and plates in trabecular bone^81^. Changes in trabecular bone with age (and particularly its osteoporotic degradation) are characterised by transition from plate-like to rod-like architecture, which results in higher SMI values. (7) Degree of anisotropy (DA) determines the degree of anisotropy of trabecular bone. The values of this parameter range from 0 (total isotropy) to 1 (total anisotropy). Since more horizontal trabeculae are lost with age (and especially in osteoporosis conditions) than vertical ones, the trabecular bone increases its structural anisotropy. (8) Connectivity density (Conn.D) indicates the connection density between the trabeculae in a given sample volume.

All selected parameters describe well the alterations of the trabecular bone occurring with age, consisting of the reduction of bone quantity in a given volume of the sample, the rarefaction of the trabecular network, changes in the thickness and shape of trabeculae, an increase in the distance between trabeculae, a decrease in their connectivity and an increase in the degree of anisotropy of trabecular bone as a result of preferential loss of horizontal trabeculae with aging^19^.

### Diagenetic changes

In order to assess whether the analysed skeletal material was affected by diagenetic changes, Fourier transform infrared spectroscopy (FTIR) was performed on samples taken from selected bones. Two parameters were calculated: the crystallinity index (CI)^82^ and the carbonate/phosphate ratio (C/P)^83^. Eight femora were selected for examination from each of the Neolithic and Mediaeval series. The bone samples were purified and ground in a mill (Mixer Mill MM200, Retsch), and subsequently 1 mg of each sample was mixed with 100 mg of potassium bromide (KBr). Subsequently, FT-IR spectra were recorded for each sample using a FT-IR NEXUS spectrometer (Thermo Nicolet), and the obtained results were analysed at the Laboratory of Molecular Spectroscopy, Department of Organic Chemistry, University of Lodz, according to published methodology^84^. Almost all the examined samples fell within ranges of CI and C/P that are typical for unaltered bones (C/P between 2.5 and 3.5, and CI between 0.31 and 0.65)^85–87^. Only two specimens from the Neolithic series exceeded the normal C/P range (0.68 and 0.71). Since the differences were slight and not consistent with diagenetic changes, and the CI values for those specimens were within the norm, they were not excluded from further analyses.

### Statistical methods

Each variable was preliminarily evaluated for homogeneity of variances using the Shapiro Wilk test. Correlation between each trabecular bone architecture parameter of vertebrae and biological age was examined with Spearman’s rank correlation coefficient^88,89^. Trabecular bone parameters variation across skeletal series and age groups was evaluated by means of two-way ANOVA after Box-Cox transformation of each variable. A multifactorial regression model was used in order to evaluate the relationship between trabecular bone architecture parameters and: analysed series (Neolithic, Mediaeval), biological sex (male, female), individual’s age at death and stature. In Two-Way ANOVA and regression analysis, the number of the cases was reduced to 56 skeletons with the presence of all given analysed features.

Bonferroni corrections to the *p* values both in ANOVA and multivariate analysis have been calculated. Because both analyses have been repeated three times, so the corrected p values were 0.0125.

Statistical analysis was performed using the STATISTICA 13.0 software.

Moreover, it should be underlined that the other investigated parameters (Tb.Th, Tb.Pf, SMI, DA) showed no statistically significant differences and were therefore omitted from the present analysis.

### Ethical approval

For this study the authors followed the Polish national regulations and laws for the analysis of archaeological human remains. Permission to study the remains was issued by the directorates of the Museum of Archaeology and Ethnography in Lodz, by the Department of Anthropology and by the Faculty of Biology and Environmental Protection of the University of Lodz (Poland).

## Results

### Pathological and diagenetic changes

Bone lesions were found in 19.4% of individuals (12 out of 62 examined). In 10 individuals, the lesions concerned the spine and included osteophytes, Schmorl’s nodes, and a case of osteochondrosis in the cervical region. The remaining three cases were moderate degenerative changes on the articular surface of the femoral heads (one individual suffered from lesions in both spine and hip joint). A relatively common condition in the examined skeletal sample was sacralisation of the fifth lumbar vertebra, which was found in seven individuals. The above-mentioned spine lesions were much common in the Neolithic individuals (6/19, 31.6%) than in the Mediaeval ones (4/43, 9.3%) (chi-square = 4.83, *p* < 0.05). This difference cannot be explained by the age of the individuals, because the frequencies in the older age classes in both samples are similar. The same direction of differences between the analysed periods is shown by the frequency of sacralisation of the fifth lumbar vertebra: 26.3% (5/19) in the Neolithic and 4.7% (2/43) (chi-square = 6.18, *p* < 0.05). In the Mediaeval sample, all spine pathologies occurred only in men (4/20, 20.0%), while in the Neolithic sample they affected both sexes (males: 3/12, 25%; females 3/7, 42.9%). Sex differences did not occur in the studied samples in the case of sacralisation of the lumbar vertebra. As expected, the observed spine lesions are positively related to the age of the individual: for both sexes together, their frequency in the 18–29 age group is 4.3%, in the 30–49 age group 17.4%, and in the 50 + age group 31.3%. This relationship also occurs for each sex separately.

Values determining diagenetic changes of the analysed skeletons: the crystallinity index (CI) and the carbonate/phosphate ratio (C/P) in all examined samples were within the ranges calculated for unaltered bones^6^.

### Trabecular architecture analysis

**Figure 3:**
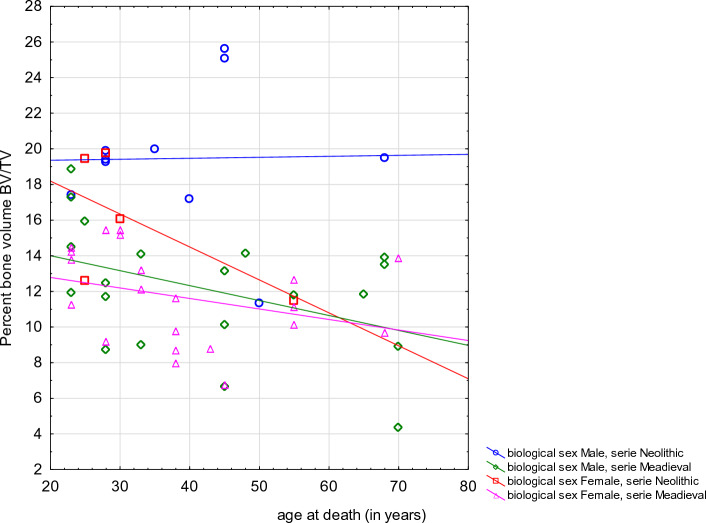
Scatter plot of the percent bone volume (BV/TV) against the reconstructed age at death of the analysed individuals by sex and population (Spearman’s rank correlation **R =  − 0.3470, *p* = 0.008**).

**Figure 4:**
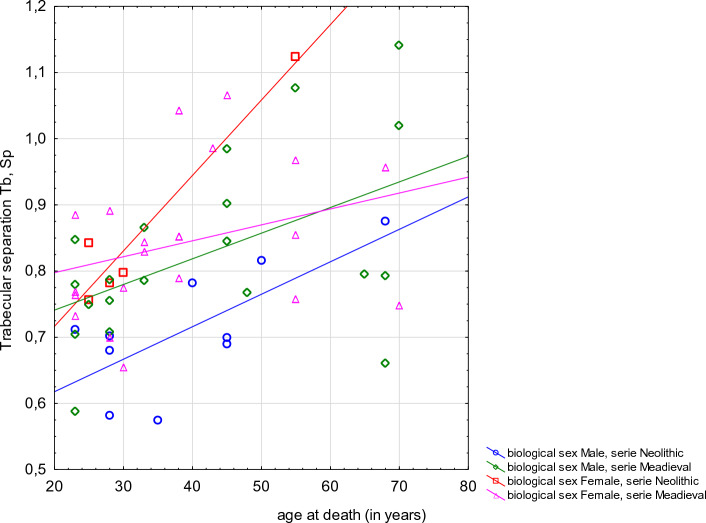
Scatter plot of the trabecular separation (Tb.Sp) against the reconstructed age at death of the analysed individuals by sex and population (Spearman’s rank correlation **R = 0.4247, *p* = 0.001**).

**Figure 5:**
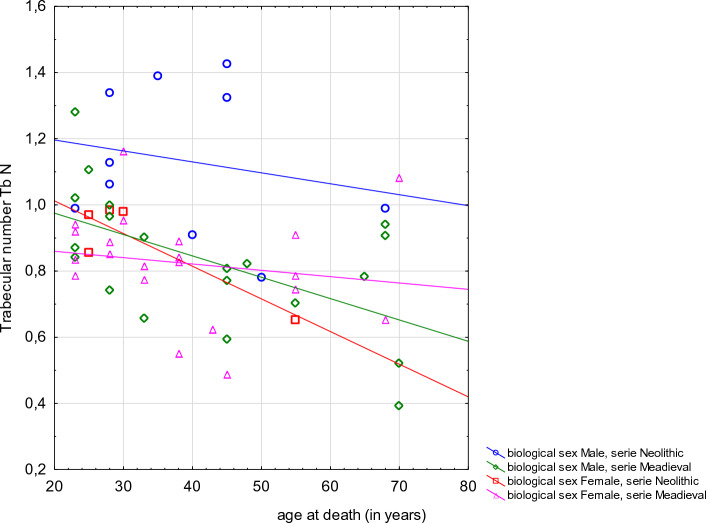
Scatter plot of connectivity density (Conn.D) against the reconstructed age at death of the analysed individuals by sex and population (Spearman’s rank correlation **R = **− **0.3111, *p* = 0.019**).

CT analysis of vertebrae in Neolithic and Mediaeval populations revealed age-related variability in trabecular bone, which is also typical of contemporary human populations. In both skeletal samples percentage bone volume, trabecular number and connectivity density were negatively correlated with biological age (results of Spearman’s rank correlation respectively: R = − 0.3470, *p* = 0.008; R = − 0.3863, *p* = 0.003; R = − 0.3111, *p* = 0.019). In both skeletal samples trabecular separation was positively correlated with biological age (R = 0.4247, *p* = 0.001). Scatter plots of BV/TV, Tb.Sp, and Tb.N against the reconstructed age at death are presented in Figs. 3, 4, 5.

In both skeletal samples correlation between trabecular thickness, trabecular pattern factor, structure model index and degree of anisotropy and biological age is insignificant (results of Spearman’s rank correlation respectively: R = − 0.1027, *p* = 0.461; R = 0.1243, *p* = 0.358; R = 0.1476, *p* = 0.274; R = 0.1340, *p* = 0.327). Further statistical analyses were performed for parameters significantly correlated with biological age. For further analyses, we also chose only one parameter measuring the number of trabeculae – Tb.N. Values of the Mann–Whitney U test for comparing the mean of the analysed parameters: percentage bone volume fraction (BV/TV), trabecular separation (Tb.Sp) and trabecular number (Tb.N) between males and females in both series were statistically insignificant.

Differences in trabecular bone parameters between Neolithic and Mediaeval samples regarding age class were analysed using two-way Anova. Because the results of the Shapiro–Wilk test for all variables point to the lack of homogeneity of variances, the Box-Cox transformation was used prior to the ANOVA analysis.

The Neolithic population was characterised by significantly higher values of percentage bone volume fraction (BV/TV), trabecular number (Tb.N)and connectivity density (Conn.D), with a statistically important interaction between series and age at death. Moreover, higher Tb.Th and lower Tb.Pf and SMI values suggest greater trabecular thickness, better trabecular connectivity, and more plate-like trabecular bone architecture (Supplementary material Table 1). These results indicate that the studied Neolithic population appears to be characterised by a significantly better condition of trabecular bone architecture than the Mediaeval population (Table 2).

**Table 2:** Average trabecular bone parameters of the fourth lumbar vertebrae in Neolithic and Mediaeval series.

Series	All individuals			18–29 years			30–49 years			50 +			F; p
	N	Mean	SD	n	Mean	SD	n	Mean	SD	n	Mean	SD	
BV/TV													
Neolithic	15	18.26	4.20	7	18.24	2.63	5	20.79	4.39	3	14.09	4.69	Age at death: **F = 3.30; *p* = 0.04**
Mediaeval	41	11.89	3.06	14	13.54	2.90	16	11.03	2.94	11	11.06	2.79	
	Series: **F = 26.79; * p* < 0.01**			Series x Age at death: F = 2.85; *p* = 0.07									
Tb.Sp													
Neolithic	15	0.76	0.13	7	0.72	0.08	5	0.71	0.09	3	0.94	0.16	Age at death: **F = 7.02; * p* < 0.01**
Mediaeval	41	0.84	0.12	14	0.76	0.08	16	0.86	0.11	11	0.89	0.15	
	Series: F = 2.51; *p* = 0.12			Series x Age at death: F = 2.93; *p* = 0.06									
Tb.N													
Neolithic	15	1.05	0.23	7	1.05	0.15	5	1.21	0.24	3	0.81	0.17	Age at death: **F = 4.67; *p* = 0.01**
Mediaeval	41	0.83	0.18	14	0.93	0.14	16	0.78	0.17	11	0.76	0.20	
	Series: **F = 10.60; *p* < 0.01**			Series x Age at death: **F = 3.59; *p* = 0.03**									
Conn.D													
Neolithic	15	10.76	6.04	7	9.64	5.17	5	14.41	7.64	3	7.30	1.52	Age at death: F = 1.53; *p* = 0.23
Mediaeval	41	6.61	3.58	14	8.48	4.36	16	5.78	2.86	11	5.45	2.63	
	Series: **F = 9.47; * p* < 0.01**			Series x Age at death: F = 2.36; *p* = 0.11									

Results of two-way ANOVA (dependent variables after Box–Cox transformation) highlighting differences between the series and between ages at death.

Significant values are in bold.

All results except age at death in analysis of percentage bone volume fraction and the interaction between series and age at death in analysis of trabecular number remained significant after applying Bonferroni corrections of *p* values.

Results of multiple regression analysis where the dependent variable is selected trabecular bone parameter and independent variables are: series (Neolithic, Mediaeval), biological sex (female, male), biological age and stature, were presented in Table 3. Multiple regression analysis showed that from 24–48% of the variability of trabecular architecture parameters was explained by the following factors taken together: the skeletal series of the specimen, the individuals’ age at death, biological sex and stature. Adjusted R^2^ were as follows: for percentage bone volume fraction R^2^ = 0.48, for trabecular separation R^2^ = 0.25, for trabecular number R^2^ = 0.32 and for connectivity density R^2^ = 0.24. The slopes of regression lines indicate that: BV/TV significantly decreased with age at death and decreased with chronological series (Mediaeval vs Neolithic); Tb.Sp significantly increased with age at death; Tb.N significantly decreased with age at death and is lower in later Mediaeval series; Conn.D is significantly higher in the Neolithic population.

**Table 3:** Results of multiple regression analysis of trabecular bone variability of the fourth lumbar vertebrae according to skeletal series, biological sex, individual’s age at death and stature (Pearson’s reconstructed stature).

Variable	Beta	SE Beta	B	SE b	t	p
BV/TV: R = 0.71; R^2^ = 0.50; adjusted R^2^ = 0.46; F = 12.85; *p* < 0.01						
			44.37	20.86	**2.13**	**0.04**
Series^a^	− 0.55	0.11	− 5.46	1.06	− **5.16**	**0.00**
Sex^b^	− 0.39	0.22	− 3.42	1.94	− 1.77	0.28
Age at death	− 0.24	0.10	− 0.07	0.03	− **2.38**	**0.02**
Stature	− 0.24	0.22	− 0.14	0.13	− 1.11	0.27
Tb.Sp: R = 0.57; R^2^ = 0.33; adjusted R^2^ = 0.27; F = 6.16; * p* < 0.01						
			− 0.35	0.71	− 0.53	0.62
Series	0.11	0.12	0.03	0.04	0.91	0.37
Sex	0.56	0.26	0.14	0.07	**2.16**	**0.04**
Age at death	0.46	0.12	0.00	0.00	**3.95**	**0.00**
Stature	0.34	0.25	0.01	0.00	1.34	0.19
Tb.N: R = 0.60; R^2^ = 0.37; adjusted R^2^ = 0.32; F = 7.35; * p* < 0.01;						
			2.95	1.16	**2,55**	**0.01**
Series	− 0.34	0.12	− 0.16	0.06	− **2.77**	**0.01**
Sex	− 0.52	0.25	− 0.23	0.11	− **2.93**	**0.04**
Age at death	− 0.33	0.11	− 0.00	0.00	− **2.61**	**0.01**
Stature	− 0.36	0.25	− 0.01	0.01	− 1.47	0.15
Conn. D: R = 0.55; R^2^ = 0.31; adjusted R^2^ = 0.25; F = 5.65; * p* < 0.01;						
			36.45	26.38	1.38	0.18
Series	− 0.28	0.13	− 2.97	1.33	− **2.23**	**0.03**
Sex	− 0.51	0.26	− 4.75	2.43	− 1.95	0.06
Age at death	− 0.25	0.12	− 0.08	0.04	− **2.13**	**0.04**
Stature	− 0.20	0.26	− 0.13	0.16	− 0.79	0.43

Significant values are in bold.

^a^Series codes: 0—Neolithic, 1—Mediaeval.

^b^Biological sex codes: 0—males, 1—females.

After using Bonferroni correction of p values, the slopes of regression lines indicate that: BV/TV still significantly decreased with chronological series; Tb.Sp significantly increased with age at death; Tb.N significantly decreased with age et death and is higher in the Neolithic population.

## Discussion

Trabecular bone has a characteristic network of lamellar bony plates and rods which shows less density, homogeneity and lower degree of parallel orientation. It was thought to receive its blood supply through a mechanism of diffusion from the surrounding bone marrow, although large and wide trabeculae may have blood vessels in them, while small trabeculae are surrounded by vessels that send capillaries that supply the osteocytes.

It shows a wide variability in strength and stiffness to be related with apparent density of this type of bone^10^. Osteoporotic bone loss occurs as a result of an imbalance of the remodelling process governing bone homeostasis, noting that this remodelling activity is higher in the central skeleton, which explains bone loss-related vertebral fractures^10^. Although trabecular bone accounts only for approximately 20% of the total skeletal bone mass, it has often been considered responsible for most of a skeleton’s turnover, which is particularly apparent in individuals younger than 65 years of age^10^, although such a view was questioned in the past by Parfitt^90^.

In our study, four of the analysed parameters show a significant relationship with age: BV/TV, Tb.N, Tb.Sp, and Conn.D. Changes in these parameters with age are quite logically interconnected: a decrease in the amount of bone volume represents the result of a decrease in the number of trabeculae (unless they decrease in the thickness), an increase in the space between the trabeculae and a reduction in the number of their connections. The observed changes seem to relate more to amount of cancellous bone than to the form of the trabeculae themselves.

The significant decrease in BV/TV with age at death observed in our sample is compatible with data from the current biomedical literature, especially as described by Chen and colleagues, who reported on a cadaveric microCT study on n = 56 L4 vertebrae from an Asian population^91^. When assessing the relative contribution of BV/TV—a parameter that, unlike bone mineral density (BMD) or bone mineral content (BMC), only evaluates trabecular bone—to the mechanical behaviour of human vertebrae, it should be underlined that in younger populations trabecular bone contributes significantly to energy dissipation, while in elderly populations, in which a low BV/TV can be seen, cortical bone plays a greater role in the process^24^. Hence this parameter, together with BMC, BMD and vBMD (volumetric bone mineral density), is described in the literature as strongly correlated with compressive stiffness of vertebrae and failure load^24^.

Comparable data about BV/TV are to be found in a microCT study by Thomsen and colleagues^21^ that examined the L2 body from 40 female individuals and 39 male individuals.

It showed that the mean trabecular bone fraction was significantly diminished with increasing age in both sexes, without significant differences between the two sexes^21^. Our data are also in agreement with those produced in the study based on 26 autopsy cases by Grote and colleagues^92^, who confirmed a decrease in this fraction in cases aged more than 45 years at the moment of death and also from the third to the eighth decade of life^92^.

Grote and colleagues, just as previously Amling and colleagues^93^, were also able to show that the decrease in BV/TV was not the same in the various segments of the vertebral column, in that it was “*less pronounced in cranial than in caudal regions of the spine*”, hence making the lumbar spine the most affected segment (52.8% from the 3rd to 8th decade)^92^.

Regarding trabecular separation (Tb.Sp), in our sample it increased with advancing age which is in accordance with the data from the Chen et al. study^91^. As far as and Tb.N is concerned, we found that it significantly decreased with age, as also described by Chen and colleagues^19^. Such a decrease was also described by Amling and colleagues who examined 26 normal spines from autoptic cases (13 males, 13 females) and concluded that “*there was an age-related decrease of the trabecular number from the 30*^*th*^* year of age to the 80*^*th*^* year of age of 34.6%*”^93^. Chen et al.^91^ underlined a similar pattern of decrease in Tb.N in both sexes, whereas Thomsen and colleagues^21^ found this process to be much faster in females than in males, showing how the former sex might be more eminently affected by trabecular bone loss. The three parameters (decreasing BV/TV and Tb.N, increasing Tb.Sp) are thus in accordance with the existing biomedical literature when increasing age is taken into account, and, from the biological anthropological perspective, they also show a similar trend comparing a Neolithic and a later Mediaeval population.

Finally, connectivity density (Conn.D) is crucial in the process of maintenance of bone strength and known to significantly decrease with age in the modern literature and to be correlate with Tb.N in the same investigates skeletal region^91^.

When there is a decrease in trabecular bone volume, a contextual decrease in Conn.D occurs because of the likely loss of small interconnecting trabeculae^91^. In both our analysed skeletal samples connectivity density was negatively correlated with biological age. This trend was also visible in the ANOVA analysis. In our sample it was also possible to show that Conn.D is significantly higher in the Neolithic population than in the Mediaeval one.

It is often emphasised that age-related changes in bone mineral density and trabecular structure in the past were observed in younger age than in present-day populations^2,27^. Although small numbers and population heterogeneity of the analysed skeletal series make similar analysis difficult, such a phenomenon can also be observed in our sample. Comparison of the mean values of BV/TV, Tb.Sp and Tb.N (three parameters that changed significantly with age: Table 2) in the Mediaeval sample (for both sexes combined) shows much larger differences between the youngest and the middle age group, than between the middle and the oldest group. For example, BV/TV decreased by 18.5% between the 18–29 and 30–49 age groups but did not change between the 30–49 and 50 + groups. Similarly, Tb.Sp increased by 13.2% (between 18–29 and 30–49) and 3.5% (between 30–49 and 50 +), and Tb.N decreased by 16.1% (between 18–29 and 30–49) and 2.6% (between 30–49 and 50 +).

Our study indicates that the analysed Neolithic population is characterised by a significantly better condition of trabecular bone architecture than the Mediaeval population. As mentioned above we obtained similar result for bone mineral density in our previous studies of these populations. We explained the better bone maintenance in the Neolithic population, among other things, by the greater biomechanical load on the skeleton of people of that time. The same may be evidenced by the much higher frequency of the spine lesions such as osteophytes, Schmorl’s nodes and osteochondrosis in the Neolithic series found in this study. The etiology of these lesions is associated with excessive load on the spine. This could also contribute to maintaining “better” (in terms of resistance to biomechanical loads^94^) bone quality, i.e. stronger trabecular bone architecture of the vertebrae in this population. Trabecular bone architecture (of the humeral head) in Neolithic humans was also studied by Scherf and co-authors^45^, who found higher values of BV/TV, Tb.N, Tb.Th, Conn.D and DA, and lower Tb.Sp compared to modern population. Other authors^2,27^ also point to generally better trabecular bone connectivity and lifelong bone maintenance in archaeological populations from Mediaeval England and ancient Rome compared to modern populations. They all attribute this to, among other things, higher levels of physical activity in ancient populations compared to modern times. Indeed, the significance of mechanical strain and physical activity for bone formation is well documented, especially during growth, when it largely determines peak bone mass, and it remains also relevant during adulthood^95–97^. Also, in the case of skeletal series we studied, differences in physical labour seem to be the best explanation for better bone maintenance in the Neolithic population. Both Neolithic and Mediaeval populations lived in the same area, under similar bio-geographic conditions, and both were agricultural communities. However, different levels of technological advancement of these societies certainly resulted in significant differences in the physical workloads in each of them. The Neolithic BKG farmers relied solely on manual work and their subsistence required high labour inputs to ensures sufficient productivity. The use of cattle as draft animals for farming appeared only several hundred years later, in the Funnel Beaker culture^98^. Other works included clearing forests, hunting, fishing, gathering, and building settlements consisting of monumental longhouses and wooden fortifications surrounding the settlement^72^. The great physical effort required by the lifestyle of BKG farmers is also evidenced by the results of the study on musculoskeletal stress markers (MSM), which showed more pronounced muscle attachment sites in Neolithic skeletons compared to historical populations from this same region^99^.

One may wonder whether the observed better trabecular bone maintenance in the Neolithic population does not contradict the poor nutritional status of early farmers resulting from a shortage of animal proteins in the diet and excessive reliance on grains (which additionally has an inhibitory effect on calcium absorption due to high phytate content^100,101^), often emphasised in the literature^102–106^. Vitamin D and calcium deficiency could affect bone growth during childhood and adolescence, impair peak bone mass achieved in early adulthood, and bone maintenance later in life. However, the subsistence of the Neolithic BKG populations was more diverse, including crop cultivation and animal husbandry, as well as hunting, fishing and gathering^72^, to cover their nutrient and caloric demand. Moreover, from BKG culture come the earliest evidence in this part of Europe of cheese production from milk^107^, the consumption of which in its raw state was probably still limited due to lactose intolerance caused by the low frequency of mutation determining lactase persistence in adult life^108^. The greater exposure to sunlight of people in the Neolithic population compared to the Middle Ages may also have been an important factor. This resulted, on the one hand, from the Holocene climatic optimum lasting in Europe from the second half of the seventh millennium to about the middle of the fourth millennium BCE, which was characterised by higher air temperatures and longer periods of insolation^109^. On the other hand, there were probably also cultural factors causing greater exposure to sunlight for people in this population. For example, archaeological data locates all craftsmen’s workshops outside houses, so most of the activities of the inhabitants of these Neolithic settlements took place outdoors. All this could have had a beneficial effect on bone metabolism in the Neolithic population through the production of vitamin D3.

There are several studies^110–112^ indicating the relationship between trabecular bone architecture and vertebral osteophytes, osteochondrosis and intervertebral disc degeneration based on clinical data. A similar study on archeological skeletons^3^ found an association of osteophytes only with BMC of cortical bone of the vertebral body. To exclude potential impact of these conditions on the trabecular structure, we selected individuals without advanced spinal lesions. The cases of moderate pathology in older individuals that were included in the analysis can be considered a normal part of the aging process. Clinical data indicate that osteophytes even provide additional protection for the vertebrae, increasing their resistance to fractures, thus it can be said that they are adaptative rather than degenerative in nature from the point of view of biomechanics of the aging spine^113,114^.

Unlike several data from the literature, in our populations sex is a factor that significantly differentiates trabecular bone architecture. After excluding the influence of series, age and body size, men are characterised by higher Tb.N and lower Tb.Sp values compared to women. A smaller number of trabeculae and a greater distance between them without significant differences in BV/TV could suggest an increase in the thickness of the remaining trabeculae in women, as postulated by some authors (reviewed in Thomsen et al.^20^). However, the scatterplot for Tb.Th (Fig. 1 Suppl. Mat.) shows no tendency for differences in trabecular thickness between men and women in both series. Moreover, the analysis of scatterplots of individual trabecular bone parameters indicates that it is mainly Neolithic men who stan out the most in both series. This is visible both in the case of parameters that differ statistically significantly between sexes (the have the highest Tb.N values and the lowest Tb.Sp values), and where there are no significant differences (for instance, they also have the highest BV/TV and Conn.D values). This result is consistent with the above-mentioned study by Scherf and colleagues^45^ on the trabecular bone of the humeral head, who showed higher values of BV/TV, Tb.N, Tb.Th, Conn.D and DA, and lower Tb.Sp of the trabecular architecture in the Neolithic population compared to modern data in general, and in Neolithic men in particular compared to men from the modern sample. The authors concluded that the primary cause of differences in trabecular architecture of the analysed populations was the much greater physical workload of people in the Neolithic compared to the present day. Thus, our results are evidence of a higher physical workload of Neolithic farmers also compared to agricultural populations in the Middle Ages.

The lack of sex differences in bone maintenance in the rural Mediaeval population of Wharram Percy was explained by Agarwal and colleagues^27^ by equally strenuous lifestyles of rural men and women. Following this interpretation, such differences in physical workload between sexes occurred in the populations analysed in our study. Another explanation requiring some discussion concerns the reproductive factor. As mentioned above, clinical data indicate that parity and breastfeeding may temporarily reduce bone mass in women. It is estimated that decrease in bone mineral density may reach 3% during pregnancy and 4–7% during lactation^115^. On the other hand, the only work so far verifying the potential impact of pregnancy and lactation on women’s BMD based on a series of historical skeletons from individuals of known parity status showed no such relationship^116^. For this factor to be responsible for the reduced parameters of trabecular bone in women from our series (and thus for sex differences in this respect), the study sample would have to include skeletons of young women who were pregnant, shortly after giving birth or during breastfeeding at the time of death. Based on the available data, we cannot verify this hypothesis, but the scatterplots should in such a situation show low values of trabecular bone parameters in women in younger age group and rather no clear tendency for their changes later in life. We did not notice such a patterns of changes with age in our data, but it should be noted that the emerging trends are often based on single observations.

Regardless of the reasons for the observed sex differences, their direction, i.e. better bone quality in men (according to the definition proposed by Bouxsein as “totality of features and characteristics that influence a bone’s ability to resist fracture”^13^) is in contradiction with the higher frequency of fractures considered osteoporotic in men, that we have found in our previous studies.

As previously mentioned, the other investigated parameters (Tb.Th, Tb.Pf, SMI, DA) showed no statistically significant differences. This aspect, by itself, justifies the question whether the absence of such differences can also be regarded as a significant result. For instance, the lack of age differences in the degree of anisotropy appears to indicate that there is no loss of horizontal trabeculae (and trabecular bone loss with age in general) as is, instead, seen—it being in fact quite typical of—modern osteoporosis^19^. Perhaps this could be explained by the fact that there were no advanced changes in the trabecular structure typical of osteoporosis among the individuals studied. The fact that significant changes in the degree of anisotropy of the trabecular bone with age also occurred in the past populations are proven by the study on the Mediaeval skeletal sample from Wharram Percy, England^27^. In the case of Tb.Th the situation is more complex. Most data for contemporary populations show a reduction in the thickness of the bone trabeculae with age. This is an obvious consequence of the decreasing amount of bone tissue in the volume of the vertebral body. However, although BV/TV decreases significantly in our populations, Tb.Th does not undergo such changes (a similar result was obtained for the above-mentioned Mediaeval population from Wharram Percy). An explanation here may be the results presented by Thomsen et al.^21^ showing that the thickness of horizontal and vertical trabeculae changes unequally and a better measure of changes with age is the ratio of the thickness of both groups to each other. In our study, it was not possible to separate Tb.Th values for horizontal and vertical trabeculae.

The lack of significant changes in Tb.Pf suggests no deterioration of the connectedness of the trabecular structure of the examined vertebrae with age. However, in the light of other studies in which this parameter was subject to significant changes with age, this may be more of a problem of the reliability of Tb.Pf as an indicator of connectivity. In present study Tb.Pf was selected as a standard analysis element offered in the tomograph software package^27^.

## Conclusions

The changes with age in trabecular architecture observed in ancient and historical populations from north-central Poland are generally consistent with data from the current biomedical literature^117^.

The Neolithic population is characterised by a significantly better condition of trabecular bone architecture than its Mediaeval counterpart. Future studies of past populations should try to further investigate, using microCT, the relations between loss of vertical vs horizontal trabeculae, confirming or disproving what we preliminarily highlighted in the present study. Additional analyses of this kind, potentially run multicentrally and aptly correlated with current clinical data could help science understand the exact evolutionary processes that led to the establishment of the nosological entity of osteoporosis.

### Supplementary Information

Supplementary Legends.

Supplementary Figure 1.

Supplementary Figure 2.

Supplementary Figure 3.

Supplementary Figure 4.

Supplementary Figure 5.

Supplementary Table 1.

## Data Availability

Data available on request from the authors. The data that support the findings of this study are available from the corresponding author upon reasonable request.
