# Supplementary material for: Age- and sex-related changes in vertebral trabecular bone architecture in Neolithic and Mediaeval populations from Poland
Source: Sci Rep. 2024 May 1;14:9977. doi: 10.1038/s41598-024-59946-z (PMC11063184; doi:10.1038/s41598-024-59946-z)
Supplement: Supplementary file 1 — Supplementary Legends. [file 41598_2024_59946_MOESM1_ESM.docx]

**Supplementary Material Legends**

Scatter plots of features insignificantly correlated with age at death against the reconstructed age at death of the analysed individuals by sex and population are presented in *Supplementary material* (Fig. 1-4).

Supplementary material Figure 1. Scatter plot of the trabecular thickness (Tb.Th) against the reconstructed age at death of the analysed individuals by sex and population (Spearman’s rank correlation R=−0.1027, p=0.66).

Supplementary material Figure 2. Scatter plot of the trabecular pattern factor (Tb.Pf) against the reconstructed age at death of the analysed individuals by sex and population (Spearman’s rank correlation R=0.1243, p=0.67).

Supplementary material Figure 3. Scatter plot of the structure model index (SMI) against the reconstructed age at death of the analysed individuals by sex and population (Spearman’s rank correlation R=0.1476, p=0.52).

Supplementary material Figure 4. Scatter plot of the degree of anisotropy (DA) against the reconstructed age at death of the analysed individuals by sex and population (Spearman’s rank correlation R=0.1340, p=0.42).

Additionally on Figure 5. scatter plot of the connectivity density (Conn.D) against the reconstructed age at death of the analysed individuals by sex and population was presented.

Supplementary material Figure 5. Scatter plot of the connectivity density (Conn.D) against the reconstructed age at death of the analysed individuals by sex and population (Spearman’s rank correlation **R=-0.3111, p<0.05**).
