# Supplementary material for: Age- and sex-related changes in vertebral trabecular bone architecture in Neolithic and Mediaeval populations from Poland
Source: Sci Rep. 2024 May 1;14:9977. doi: 10.1038/s41598-024-59946-z (PMC11063184; doi:10.1038/s41598-024-59946-z)
Supplement: Supplementary file 7 — Supplementary Table 1. [file 41598_2024_59946_MOESM7_ESM.docx]

**Supplementary Material Table 1**. Average trabecular bone parameters of the fourth trabecular bone architecture measurements in Neolithic and Mediaeval series for which correlation with age at death is statistically insignificant. Results of two-way ANOVA (dependent variables after Box-Cox transformation) highlighting differences between the series and between ages at death.

| **Series** | All individuals | | | 18–29 years | | | 30–49 years | | | 50+ | | | F; p |
| --- | --- | --- | --- | --- | --- | --- | --- | --- | --- | --- | --- | --- | --- |
|  | N | Mean | SD | n | Mean | SD | n | Mean | SD | n | Mean | SD |  |
| Tb.Th |  |  |  |  |  |  |  |  |  |  |  |  |  |
| Neolithic | 15 | 0.17 | 0.02 | 7 | 0.18 | 0.02 | 5 | 0.17 | 0.02 | 3 | 0.17 | 0.03 | Age at death:  F=0.10; p=0.91 |
| Mediaeval | 41 | 0.14 | 0.02 | 14 | 0.15 | 0.02 | 16 | 0.14 | 0.02 | 11 | 0.15 | 0.02 |  |
|  | Series: **F=19.84; p<0.01** | | | Series x Age at death: F=0.02; p=0.98 | | | | | | | | |  |
| Tb.Pf |  |  |  |  |  |  |  |  |  |  |  |  |  |
| Neolithic | 15 | 5.01 | 2.18 | 7 | 5.15 | 1.82 | 5 | 4.30 | 2.43 | 3 | 6.31 | 2.77 | Age at death:  F=0.67; p=0.52 |
| Mediaeval | 41 | 7.30 | 1.96 | 14 | 7.34 | 2.57 | 16 | 7.42 | 1.73 | 11 | 7.09 | 1.51 |  |
|  | Series: **F=11.37; p<0.01** | | | Series x Age at death: F=1.17; p=0.32 | | | | | | | | |  |
| SMI |  |  |  |  |  |  |  |  |  |  |  |  |  |
| Neolithic | 15 | 1.31 | 0.40 | 7 | 1.35 | 0.25 | 5 | 1.09 | 0.55 | 3 | 1.56 | 0.38 | Age at death:  F=1.36; p=0.27 |
| Mediaeval | 41 | 1.64 | 0.21 | 14 | 1.62 | 0.25 | 16 | 1.65 | 0.17 | 11 | 1.64 | 0.22 |  |
|  | Series: **F=9.61; p<0.01** | | | Series x Age at death: F=1.50; p=0.23 | | | | | |  |  |  |  |
| DA |  |  |  |  |  |  |  |  |  |  |  |  |  |
| Neolithic | 15 | 1.42 | 0.21 | 7 | 1.37 | 0.20 | 5 | 1.41 | 0.19 | 3 | 1.54 | 0.31 | Age at death:  F=0.80; p=0.45 |
| Mediaeval | 41 | 1.51 | 0.19 | 14 | 1.49 | 0.17 | 16 | 1.54 | 0.22 | 11 | 1.50 | 0.19 |  |
|  | Series: F=2.57; p=0.12 | | | Series x Age at death: F=0.57; p=0.57 | | | | | | | | |  |
