# Supplementary material for: Age- and sex-related changes in vertebral trabecular bone architecture in Neolithic and Mediaeval populations from Poland
Source: Sci Rep. 2024 May 1;14:9977. doi: 10.1038/s41598-024-59946-z (PMC11063184; doi:10.1038/s41598-024-59946-z)
Supplement: Supplementary file 4 [file 41598_2024_59946_MOESM3_ESM.docx]

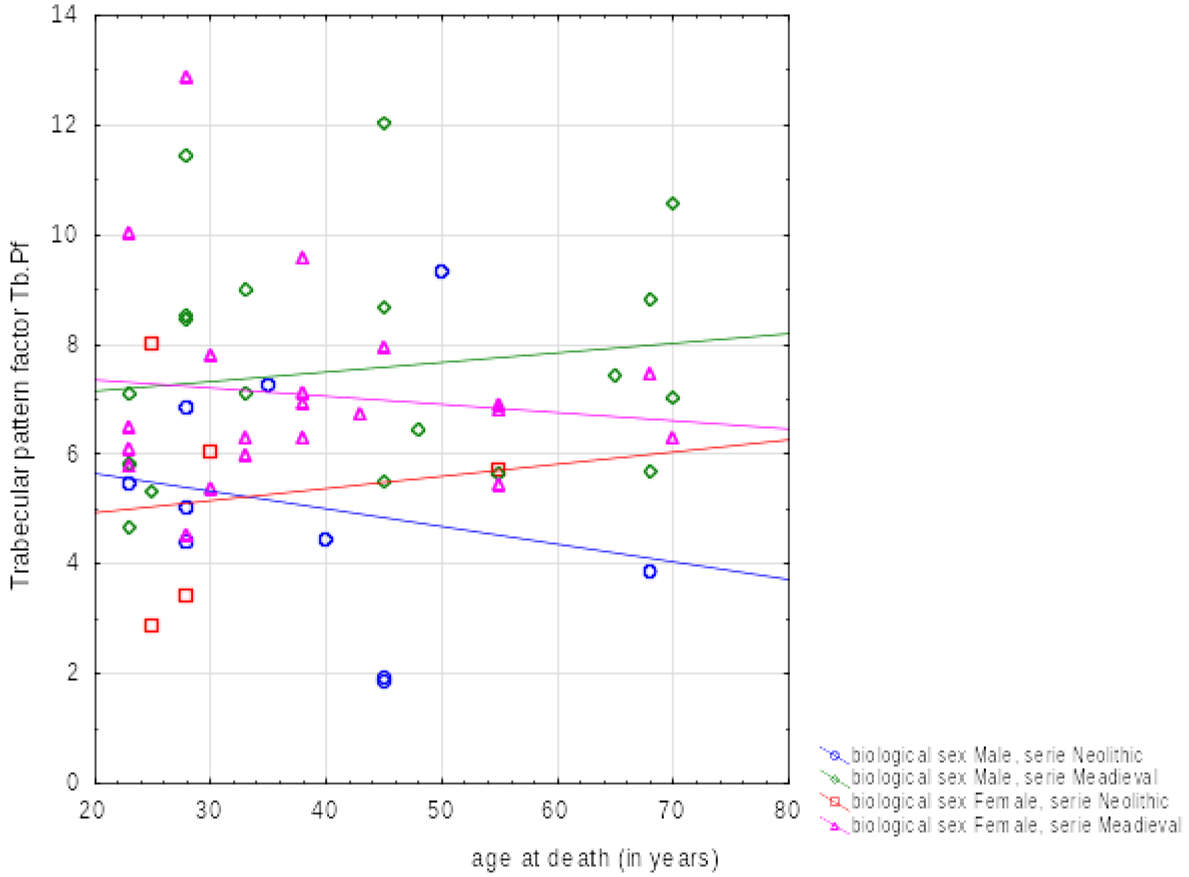

Trabecular pattern factor Tb.Pf
age at death (in years)
biological sex Male, serie Neolithic
biological sex Male, serie Meadieval
biological sex Female, serie Neolithic
biological sex Female, serie Meadieval
