# Supplementary material for: Age- and sex-related changes in vertebral trabecular bone architecture in Neolithic and Mediaeval populations from Poland
Source: Sci Rep. 2024 May 1;14:9977. doi: 10.1038/s41598-024-59946-z (PMC11063184; doi:10.1038/s41598-024-59946-z)
Supplement: Supplementary file 5 [file 41598_2024_59946_MOESM4_ESM.docx]

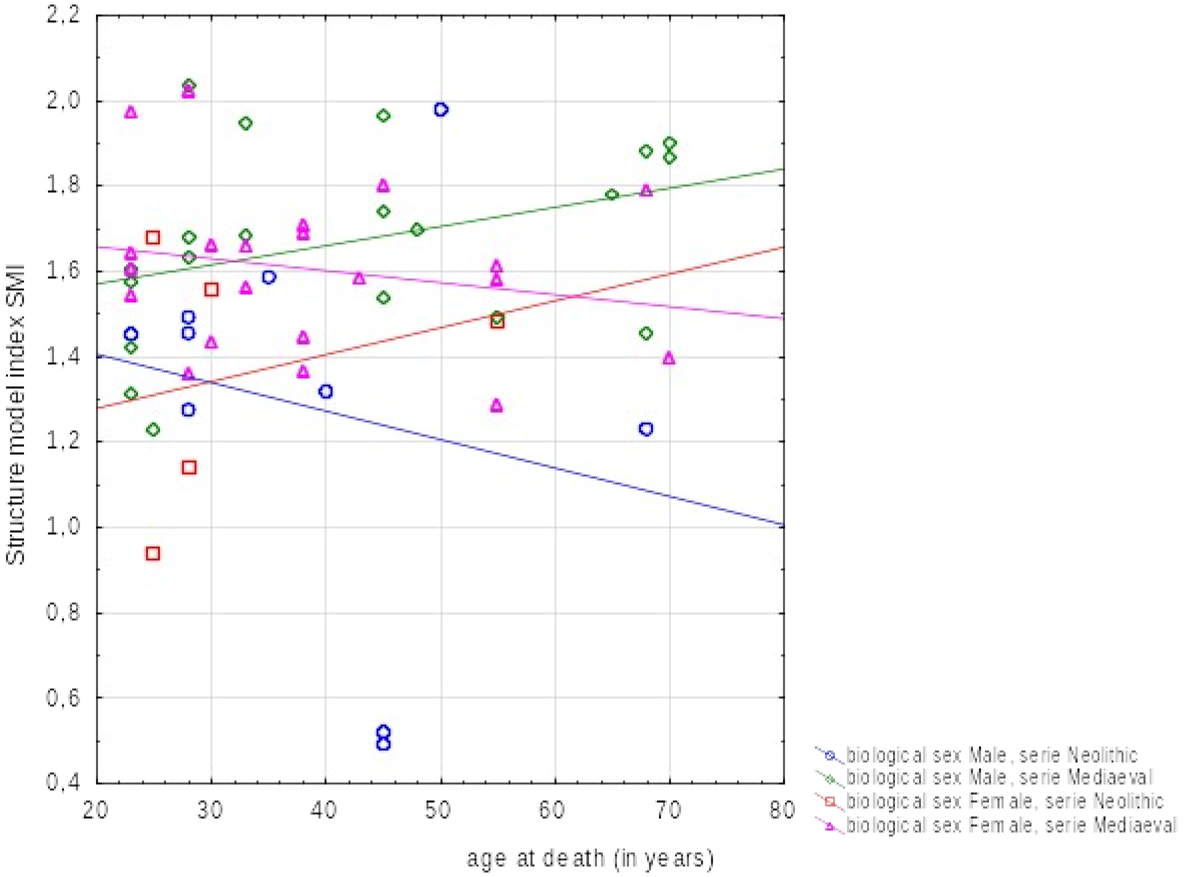

Structure model index SMI
age at death (in years)
2,2
2,0
1,8
1,6
1,4
1,2
1,0
0,8
0,6
0,4
20
30
40
50
60
70
80
biological sex Male, serie Neolithic
biological sex Male, serie Mediaeval
biological sex Female, serie Neolithic
biological sex Female, serie Mediaeval
