# Supplementary material for: Age- and sex-related changes in vertebral trabecular bone architecture in Neolithic and Mediaeval populations from Poland
Source: Sci Rep. 2024 May 1;14:9977. doi: 10.1038/s41598-024-59946-z (PMC11063184; doi:10.1038/s41598-024-59946-z)
Supplement: Supplementary file 7 [file 41598_2024_59946_MOESM6_ESM.docx]

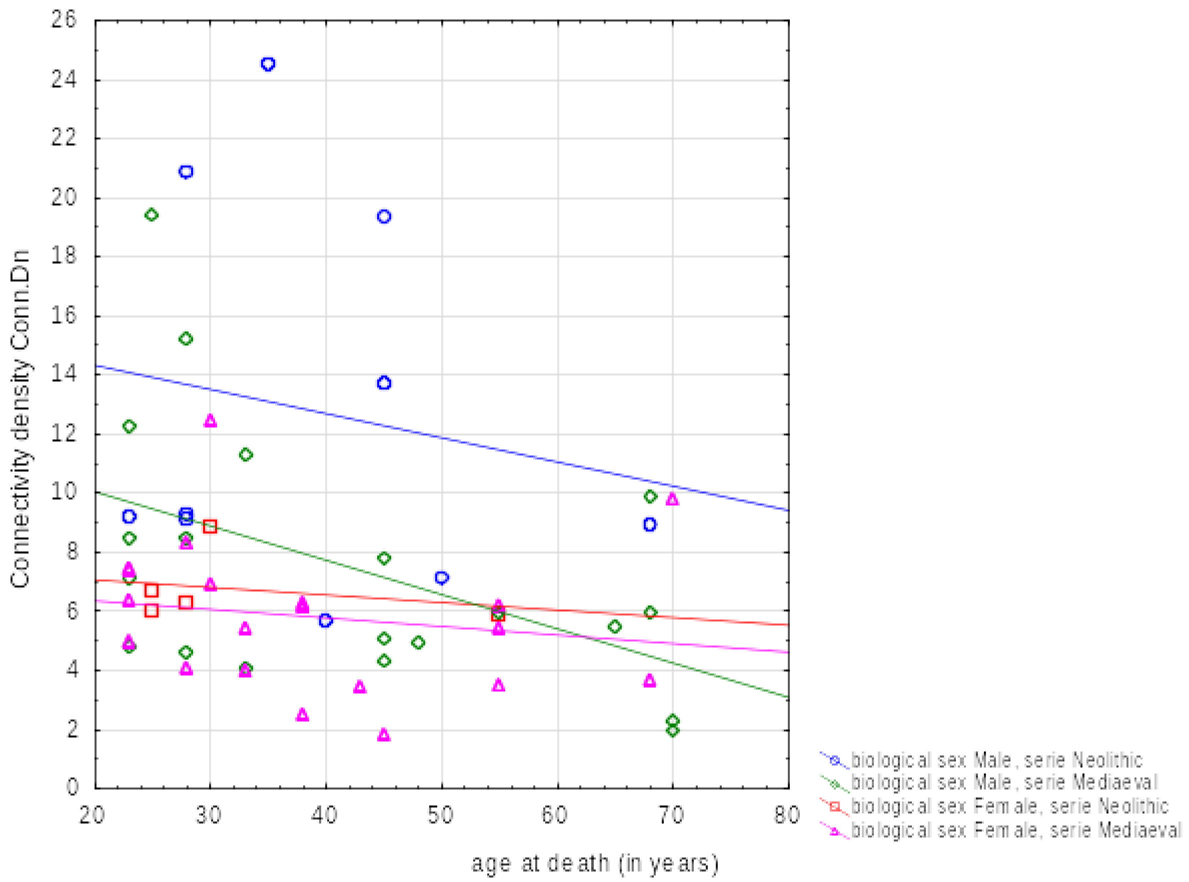

Connectivity density Conn.Dn
age at death (in years)
0
2
4
6
8
10
12
14
16
18
20
22
24
26
20
30
40
50
60
70
80
biological sex Male, serie Neolithic
biological sex Male, serie Mediaeval
biological sex Female, serie Neolithic
biological sex Female, serie Mediaeval
